# Impact Factors of Empathy in Mainland Chinese Youth

**DOI:** 10.3389/fpsyg.2020.00688

**Published:** 2020-05-19

**Authors:** Qing Zhao, Qiaoyue Ren, Yuanmiao Sun, Li Wan, Li Hu

**Affiliations:** ^1^Department of Pain Management, The State Key Clinical Specialty in Pain Medicine, Second Affiliated Hospital of Guangzhou Medical University, Guangzhou, China; ^2^CAS Key Laboratory of Mental Health, Institute of Psychology, Chinese Academy of Sciences, Beijing, China; ^3^Department of Psychology, University of Chinese Academy of Sciences, Beijing, China

**Keywords:** empathy, medical training, Interpersonal Reactivity Index, Classification and Regression Tree analysis, personal distress

## Abstract

Empathy was investigated in 592 Mainland Chinese youth using the Interpersonal Reactivity Index. Participants’ empathy-related information covering demographic traits, emotional wellness, as well as academic and social problems were recorded. Results of Classification and Regression Tree (CART) analysis showed that emotional empathy, cognitive empathy, and empathy-related personal distress was impacted by inherited traits (e.g., sex), acquired traits (e.g., study major), and a combination of both aspects, respectively. Moreover, empathy was found to be higher in youth in a vulnerable social position (i.e., outlander, female, and ethnic minority) than those in a dominant one (i.e., local, male, and ethnic majority). It was also found that personal distress, rather than empathy, was significantly correlated with academic, social, and emotional problems in the youth cohort. Hence, the current study provided an innovative observation of the relationships between empathy, personal distress, ethnicity, social vulnerability, wellness, study major, and other key characteristics in Mainland Chinese youth.

## Introduction

Empathy is defined as sharing and understanding others’ emotions (Baron-Cohen and Wheelwright, 2004). Researchers found that the individual difference in empathy varies in tandem with inherited traits, such as sex and ethnicity (Zhao et al., 2019), and with obtained traits, such as study major (Nunes et al., 2011) and sojourning experience (Cao et al., 2015). Although these traits may all have an impact on empathy, their relative importance in the impact remains unknown. The current study was conducted to address this issue with a cohort of Mainland Chinese youth.

Empathy has two main components, namely, emotional empathy and cognitive empathy (Cohen and Strayer, 1996; Lawrence et al., 2004; Shamay-Tsoory, 2011). *Emotional empathy* is an automatic procedure of sharing other’s emotions (Heyes, 2018; Hua et al., 2018), while *cognitive empathy* is a cognitive process and is to understand other’s feelings using perspective-taking (Oliver et al., 2018). Emotional and cognitive empathy represent the earlier and the later stage of empathy, respectively, and are dissociable in several aspects (Shamay-Tsoory et al., 2009). For example, researchers found that intranasal oxytocin selectively fostered emotional empathy but not cognitive empathy (Hurlemann et al., 2010). Similarly, personal distress (i.e., a concept to be introduced) was found to mediate the Australian—Mainland Chinese differences in cognitive but not emotional empathy (Zhao et al., 2019).

*Personal distress* is a self-oriented automatic aversive response to others’ suffering (López-Pérez et al., 2014); nevertheless, it is not a component of empathy which ought to be other-oriented (Batson et al., 1987). In other words, empathy urges individuals to be altruistic and caring for others, but personal distress causes individuals to withdraw from these actions, to protect themselves from being emotionally exhausted (López-Pérez et al., 2014). It is interesting to mention that Davis (1980), the author of Interpersonal Reactivity Index (IRI; a self-report questionnaire of empathy), treated the Personal Distress subscale of IRI (i.e., IRI-PD) as a tool measuring emotional empathy; in contrast, Baron-Cohen and Wheelwright (2004), the authors of Empathy Quotient (EQ; another self-report questionnaire of empathy), clearly stated that self-oriented personal distress is a closely related concept yet not empathy *per se*. Furthermore, researchers found that personal distress score was positively correlated with emotional empathy score, but negatively correlated with the scores of cognitive empathy and overall empathy (e.g., Zhao et al., 2019). The aforementioned theoretical debate and empirical findings highlighted the importance of investigating personal distress relative to empathy.

Sex is an important impact factor of empathy (Zhao et al., 2018). It is commonly accepted that females are more empathic than males (Baron-Cohen and Wheelwright, 2004). Nevertheless, this notion was more frequently confirmed in Western populations (Groen et al., 2015) than in Mainland Chinese (Zhao et al., 2019). For Mainland Chinese, the sex difference in empathy is diminished (Zhao et al., 2018). It is reasoned that Chinese people are influenced by Confucius’ Golden Mean philosophy, which taught people to depolarize the differences between Yin-Yang or Feminine-Masculine (Zhao et al., 2019). A study based on German and Ethiopian students suggested that sex was the most crucial factor to discriminate participants into high- and low-emotional empathy cohorts (i.e., females > males) (Dehning et al., 2013). Thereby, it is interesting to investigate, if not sex, what could be the most important impact factor of empathy in Mainland China.

Ethnicity is another essential impact factor of empathy (Zhao et al., 2019). Within American ethnic groups, researchers found that Africans had a higher self-report score on cognitive empathy than both Caucasians and Chinese, but they reported no group difference between the latter two ethnicities (Teague, 2014). Meanwhile, no group difference was found among the three ethnicities for emotional empathy scores (Teague, 2014). Within a cross-cultural investigation, researchers found that Australians tended to have higher self-report emotional and cognitive empathy than Mainland Chinese; nevertheless, this cultural difference was only significant between females but not males of the two cultures (Zhao et al., 2019). The former finding (i.e., Teague, 2014) implied that an ethnic group in a more vulnerable social position could be more empathic; nevertheless, the latter finding (i.e., Zhao et al., 2019) indicated that this notion might be modulated by culture and sex. China is a multi-ethnical country, including the Chinese Han and other 55 minor ethnicites (e.g., Tibetan and Uygur), while the impact of ethnicity on empathy within the Mainland Chinese population was largely unknown.

*Hukou* is a unique social term of China, recording the administrative region of a Chinese person’s original permanent address (e.g., Beijing Hukou or Shanghai Hukou) (Chan, 2019). It is largely an inherited characteristic from parents (Colas and Ge, 2019), although a few people manage to change their Hukou after getting a job or through marriage (Fan and Huang, 1998; Qian et al., 2019). Hukou is linked to social welfare; for example, free primary education and the right to purchase a house is usually a privilege for locals (Guo, 2010; Qian et al., 2019). In addition, in the dating scene and the job market, a city local of a better-off area tends to be more popular than outlanders (Zhao and Howden-Chapman, 2010; Qian et al., 2019). Au et al. (2011) investigated the impact of Hukou on empathy but found no group difference between locals and outlanders of junior high students in Beijing. Nevertheless, the social pressure and social expectation for showing empathy might be less for junior high students than older youth groups, such as university students. Whether Hukou has an impact on the empathy of Mainland Chinese university students is a novel research question.

Study major could be another impact factor of empathy in youth (Dehning et al., 2013). Moreover, researchers are concerned that medical training may hinder the development of empathy in students (e.g., Nunes et al., 2011). Nevertheless, findings on this topic are inconsistent: Nunes et al. (2011) found that after the 1st year of medical training, Mesoamerican students had a decline in empathy for patients (measured by the Jefferson Scale of Physician Empathy, JSPE; Hojat et al., 2001). In contrast, Wen et al. (2013) found that the score of JSPE in Mainland Chinese students increased steadily throughout 4 years of medical training. Moreover, Penprase et al. (2013) showed that among American college students in Grade 2 and above, nursing students had a higher empathy score than non-medical students in general situations (measured by EQ; Baron-Cohen and Wheelwright, 2004). Importantly, Dehning et al. (2013) argued that whether the medical course harms the development of empathy depends on the social expectation on medical workers (i.e., whether people prefer empathic or professional medical workers) and on whether the medical curriculum included humanities and art courses or not. Currently, the trajectory of empathy development of medical and non-medical students in Mainland China is unclear.

In this study, the relative impact of inherited traits (e.g., ethnicity and Hukou) and obtained traits (e.g., study major and study grade) on empathy, and the relationship between empathy and youth wellness (e.g., depression and anxiety) were investigated with 592 Mainland Chinese undergraduate students. According to the definition of emotional empathy (Heyes, 2018; Hua et al., 2018) and cognitive empathy (Oliver et al., 2018), these two components of empathy could be more significantly impacted by inherited traits (e.g., sex) and acquired traits (e.g., study major), respectively. In the aspect of personal distress, as it has an intricate relation with both emotional empathy (e.g., López-Pérez et al., 2014) and cognitive empathy (e.g., Zhao et al., 2019), the impact of inherited and acquired traits could both be significant.

## Materials and Methods

### Participants

Undergraduate students from grade 1 to grade 5 of college were recruited from four universities, located in three cities of China (viz., Beijing, Tianjin, and Dalian), including two medical universities and two comprehensive universities. E-posters of this study were broadcast on the university websites. In addition, introductions of this study were orally presented by experimenters during class breaks. Participants were informed that the current study was restricted to those who satisfied all of the following criteria: (1) Chinese nationality; (2) undergraduate student; (3) without any history of mental, cardiac, or neurological illness.

This study was a paper-pen based investigation, and questionnaires were presented to participants as a testing booklet. The questionnaires included in the booklet were the demographic sheet, Interpersonal Reactivity Index (IRI) (Davis, 1980), State-Trait Anxiety Inventory (STAI) (Spielberger, 2010), Beck Depression Inventory (BDI) (Beck et al., 1996), and Adolescent Self-Rating Life Events Check List (ASLEC) (Liu et al., 1997). Participants could choose to answer the booklet in groups or finish them alone. Moreover, participants were instructed to answer all questions and to double check before submission. All participants gave their informed consent according to a protocol proved by the last corresponding author’s institute. RMB¥30 (about US$5) was prepared for each participant as compensation.

The study sample size was calculated using Statulator^1^ (Dhand and Khatkar, 2014). With reference to both Song and Shi (2017) and Zhao et al. (2018), a reasonable sample size for the current investigation was between 132 and 668. In total, 606 participants finished the testing booklet. Among them, six were against the inclusion criteria, namely, an overseas Chinese resident and five students with a history of mental, cardiac, or neurological illness. After excluding these six participants, the missing values of the self-report item scores were examined. There were six missing values for six different items of STAI. These items’ missing values were replaced by the mode of each item. After the missing replacement, the distribution of self-report scales was examined. The BDI score was highly right-skewed, and as a result, the logarithm of the BDI was calculated to use in the following analyses. In addition, eight univariate outliers (*z*-scores > 3.29) were identified, namely, five outliers for the Empathic Concern subscale of the IRI (IRI-EC), two outliers for the State Anxiety subscale of the STAI (STAI-SAI), and one outlier for the Academic Stress subscale of the ASLEC (ASLEC-AS). These eight cases with the univariate outlier were excluded, and finally, 592 participants remained for the following analyses.

### Measures

#### Demographic Information Questionnaire

Participants were asked to finish a demographic questionnaire. It was used to collect basic personal information, including sex, age, ethnicity, Hukou address, history of relevant illness, study major, and year of college study.

#### Self-Report Questionnaires

The final 592 participants finished the IRI (Davis, 1980), STAI (Spielberger et al., 1970), and BDI (Beck et al., 1996), and 464 out of the overall sample completed the ASLEC (Liu et al., 1997). All questionnaires were presented in simplified Chinese, and detailed descriptions of these questionnaires were provided as follows.

#### Interpersonal Reactivity Index (IRI)

Interpersonal Reactivity Index (IRI) is a self-report questionnaire of empathy (Davis, 1980, 1983). A Chinese translated version of the IRI was administered in the current study. This Chinese version of the IRI has four subscales (22 items in total), namely, perspective-taking (IRI-PT; 5 items), empathic concern (IRI-EC; 6 items), personal distress (IRI-PD; 5 items), and fantasy (IRI-FS; 6 items) (Chan, 1986). Each item is rated on a 5-point Likert scale ranging from 0 (*does not describe me well*) to 4 (*describes me very well*). The range of the subscale score for IRI-PT and IRI-PD was 0 to 20, and for IRI-EC and IRI-FS was from 0 to 24.

According to Davis (1980), IRI-PT, IRI-EC, and IRI-PD were included in the current study to measure cognitive empathy, emotional empathy, and personal distress, respectively. In contrast, the IRI-FS was excluded from the following analysis, as it measures one’s tendency to appreciate the feelings of imaginary characters via reading a novel or watching a movie (Davis, 1980), rather than the interpersonal response of empathy (Baron-Cohen and Wheelwright, 2004). Based on the current sample, the Cronbach’s αs for IRI-PT, IRI-EC, and IRI-PD were 0.77, 0.53, and 0.79, respectively. These Cronbach’s α values of IRI-PT and IRI-PD were similar to the previous reports, including both the original study of IRI (Davis, 1980) and these based on Mainland Chinese participants (e.g., Zhao et al., 2018, 2019); nevertheless, the Cronbach’s α value of IRI-EC was slightly lower than these previous findings.

#### State-Trait Anxiety Inventory (STAI)

The STAI includes two subscales, namely, state anxiety (STAI-SAI) and trait anxiety (STAI-TAI), to evaluate one’s state and trait anxiety, respectively (Spielberger, 2010; Spielberger et al., 2010). In total, there were 40 items divided equally to the two subscales. Each item of STAI-SAI is rated on a 4-point Likert scale ranging from 1 (*not at all*) to 4 (*very much so*), and each item of STAI-TAI is rated on a 4-point Likert scale ranging from 1 (*almost never*) to 4 (*almost always*). The range of both subscales was 20 to 80. A Chinese version of the STAI (Li and Qian, 1995) was administered in the current study. The Cronbach’s αs for STAI-SAI and STAI-TAI were 0.91 and 0.87, respectively, which were consistent with previous findings based on the Chinese population (e.g., Shek, 1988).

#### Beck Depression Inventory (BDI)

The BDI is a self-report questionnaire for measuring depression, developed by Beck et al. (1961), and then modified by Beck et al. (1996). A 13-item short version of the Chinese translation of the BDI (Shek, 1990) was administered in the current study. Each item was presented with four options (i.e., from 0 to 3), ranging from the absolute absence of to the severe presence of a symptom of depression. The total score of BDI ranged from 0 to 39. The Cronbach’s α for BDI was 0.86 based on the current sample, which was consistent with the previous reports based on the Mainland Chinese youth population (Zhang et al., 1990; Yang et al., 2014).

#### Adolescent Self-Rating Life Events Check-List (ASLEC)

The ASLEC is a 27-item self-report questionnaire for recording negative life events commonly occurring in adolescents. According to the original study (Liu et al., 1997), these items were divided into six subscales (with a cross-loading issue), namely, social interaction (ASLEC-SI; 5 items), academic stress (ASLEC-AS; 5 items), punishment (ASLEC-PS; 7 items), deprivation (ASLEC-DP; 3 items), healthy adaptation (ASLEC-HA; 4 items), and others (ASLEC-OT; 4 items). Each item is rated on a 6-point Likert scale ranging from 0 (*did not occur*), and 1 (*occurred without influence*) to 5 (*occurred with very severe influence*). In the current study, the ASLEC-SI and ASLEC-AS were included for the following analyses, and the score of both subscales (no item was cross-loading between these two subscales) ranged from 0 to 25, with a higher score reflecting more stressful life. The Cronbach’s αs for the subscales were 0.79 and 0.63, respectively. These results were consistent with previous reports based on Mainland Chinese youth (Chen et al., 2016; Zhang et al., 2016).

### Data Analysis

Independent samples *t*-tests were conducted to examine self-report differences in cognitive empathy, emotional empathy, and personal distress (i.e., IRI-PT, IRI-EC, and IRI-PD, respectively) between subgroups of the following four categories, namely, sex (i.e., male or female), Hukou (i.e., locals who were studying in home province or outlanders who were sojourning in another province), ethnicity (i.e., the majority or minority), and study major (i.e., medical or non-medical students). One-way analyses of variance (ANOVAs) were used to examine group differences among three study grades, named as *Freshman*, *Sophomore*, and *Senior* (i.e., *Freshman* represents the newly entered and Grade 1 college students, *Sophomore* represents Grades 2 and 3 college students, and *Senior* represents Grades 4 and 5 college students).

Pearson correlation coefficients between continuous variables of IRI scores (i.e., IRI-PT, IRI-EC, and IRI-PD) and self-report empathy-related traits (i.e., STAI-SAI, STAI-TAI, BDI, ASLEC-SI, and ASLEC-AS) were calculated. Only if the significance of a correlation was less than 0.001 and the absolute value of the Pearson correlation coefficient was larger than or close to 0.30, it was considered to be a statistically meaningful correlation in the current study.

Analyses of Classification and Regression Tree (CART) (Breiman et al., 1984) were conducted to examine the impact of six independent variables (i.e., age, sex, Hukou, ethnicity, study major, and study grade) on cognitive empathy, emotional empathy, and personal distress (i.e., IRI-PT, IRI-EC, and IRI-PD, respectively). The dependent variable of the CART is usually continuous (i.e., regression tree) or in the other case categorical (i.e., classification tree); the independent variables of the analysis can be continuous, categorical, or a combination of both (Lemon et al., 2003). The CART is useful to identify meaningful independent variables (e.g., age or Hukou) to categorize participants into high- and low-score groups in the aspect of a dependent variable (e.g., an empathy score) (Lemon et al., 2003).

The process of CART analysis includes four steps (Yohannes and Webb, 1999). Firstly, CART tries to identify the most effective independent variable (e.g., Hukou) that can dichotomize the overall participants into two groups in terms of high- and low-score on the dependent variable (e.g., IRI-EC). Secondly, if the above dichotomization is successful, the CART begins to identify the second most powerful factor for each branch to further dichotomize participants into subgroups. Thirdly, the dichotomization continues for each sub-branch until the regression tree is over-grown up to its maximum capacity. Fourth, a pruning algorithm is applied from the tree tips until participants within all sub-branches are just statistically homogeneous in terms of the dependent variable score. Finally, an optimal tree is formulated according to the above analyses.

It is worth mentioning that the relative location of each independent factor in the classification tree can reflect the relative importance/necessity of these factors in dichotomizing the dependent variable (Guan et al., 2008; Ochs et al., 2012). That is, either the previously identified independent factor is more effective in the dichotomization than its successive ones, or the dichotomization abilities of the later ones depend on the previous one. Therefore, if any previous factor was removed from the CART analyses, the final structure of the classification tree could be changed. Furthermore, from the root to the tips, in each branch of the classification tree, a binary independent variable could be used only once, while a continuous independent variable could be used multiple times but each time it should have a new cut-off point (Liu and White, 1994). In this study, all analyses were processed using SPSS Version 22 (IBM Corp.).

## Results

### Demographic Information

The final participants were 592 Mainland Chinese undergraduate students (34.8% males), within the age range of 16–26 (mean age = 20.24 years, *SD* = 1.89). In the aspect of study major, students were divided into the medical and the non-medical students (65.7% medical students; no missing). All medial students were clinical medical students. These non-medical students were from Psychology, Law, and other 40 study majors. In the aspect of ethnicity (as presented on the Chinese national ID card), there was one missing value and it was replaced by the mode (i.e., the major ethnicity; that is, Chinese Han). After the missing replacement, 82.6% of students were Chinese Han and 17.4% of students were minorities (i.e., 17 minority groups, such as Manchu and Mongolian).

Students’ Hukou address (i.e., the administrative region of a Chinese’s original permanent address) was compared to the administrative region of their university. If students’ Hukou and their university were in the same administrative region of China, they were coded as locals; otherwise, they were coded as outlanders. There were five missing values for the Hukou address, and these five students were recoded as outlanders (i.e., the mode). After the missing value was replaced, the current sample was composed of 39.9% locals and 60.1% outlanders. No more missing values were found for the other demographic variables.

### Group Differences in Self-Report Questionnaires

The means and standard deviations of the IRI subscales (i.e., IRI-PT, IRI-EC, and IRI-PD) are presented in Tables 1–3 for demographic subgroups (i.e., sex, ethnicity, Hukou, study major, and study grade). In the respective table, results of independent samples *t*-tests of the IRI sub-scores were presented for dichotomous variables (i.e., sex, ethnicity, Hukou, and study major), and the result of a one-way ANOVA was for the study grade (i.e., three grades: *Freshman*, *Sophomore*, and *Senior*).

**TABLE 1:** Comparison of self-report IRI-PT between subgroups of five demographic categories.

	Available and missing			Descriptive statistics for subgroups			Results of *t*-test/ANOVA				
			
Demographic	*N*	Missing	Replacing	Subgroups (*n*)	*M*	*SD*	df	*t*/*F*	*p*	*d*/η^2^	95%CI
Sex	592	0.0%	*NA*	(1) Female (386)	12.04	3.71	590	0.21	0.836	0.02	[−0.56, 0.69]
				(2) Male (206)	11.97	3.58					
Ethnicity*	591	0.2%	By mode	(1) Chinese Han (489)	11.99	3.67	590	–0.31	0.754	–0.04	[−0.91, 0.66]
				(2) Minorities (103)	12.12	3.65					
Study major	592	0.0%	*NA*	(1) Medical (389)	11.57	3.66	590	–4.10	<0.001	–0.36	[−1.90, −0.67]
				(2) Non-medical (203)	12.86	3.54					
Hukou^†^	587	0.8%	By mode	(1) Local (236)	12.24	3.72	590	1.23	0.218	0.10	[−0.22, 0.98]
				(2) Outlander (356)	11.86	3.62					
Study grade	592	0.0%	*NA*	(1) Freshman (192)	11.48	3.60	589	2.98	0.051	0.01	/
				(2) Sophomore (218)	12.28	3.75					
				(3) Senior (182)	12.26	3.58					

IRI, Interpersonal Reactivity Index; IRI-PT, the total score for IRI perspective-taking items. Hukou, a unique social term of China, records the administrative region of a Chinese’s original permanent address. For the Study grade, Freshman represents the newly entered and Grade 1 college students, Sophomore represents Grades 2 and 3 college students, and Senior represents Grades 4 and 5 college students. *There was one missing value in the Ethnicity; this missing value was replaced by the mode of ethnicity (i.e., Chinese Han). ^†^There were five missing values in Hukou; these missing values were replaced by the mode of Hukou (i.e., Outlander).

**TABLE 2:** Comparison of self-report IRI-EC between subgroups of five demographic categories.

	Available and missing			Descriptive statistics for subgroups			Results of *t*-test/ANOVA				
			
Demographic	*N*	Missing	Replacing	Subgroups (*n*)	*M*	*SD*	df	*t*/*F*	*p*	*d*/η^2^	95%CI
Sex	592	0.0%	*NA*	(1) Female (386)	17.16	3.40	590	2.18	0.030	0.19	[0.06, 1.20]
				(2) Male (206)	16.53	3.25					
Ethnicity*	591	0.2%	By mode	(1) Chinese Han (489)	16.85	3.37	590	–1.36	0.173	–0.15	[−1.21, 0.22]
				(2) Minorities (103)	17.35	3.32					
Study major	592	0.0%	*NA*	(1) Medical (389)	17.15	3.29	590	2.11	0.035	0.18	[0.04, 1.18]
				(2) Non-medical (203)	16.54	3.47					
Hukou^†^	587	0.8%	By mode	(1) Local (236)	16.49	3.47	590	–2.68	0.008	–0.22	[−1.30, −0.20]
				(2) Outlander (356)	17.24	3.26					
Study grade	592	0.0%	*NA*	(1) Freshman (192)	16.83	3.68	589	1.63	0.197	0.10	/
				(2) Sophomore (218)	16.73	3.38					
				(3) Senior (182)	17.31	2.95					

IRI, Interpersonal Reactivity Index; IRI-EC, the total score for IRI empathic concern items. Hukou, a unique social term of China, records the administrative region of a Chinese’s original permanent address. For the Study grade, Freshman represents the newly entered and Grade 1 college students, Sophomore represents Grades 2 and 3 college students, and Senior represents Grades 4 and 5 college students. *There was one missing value in the Ethnicity; this missing value was replaced by the mode of ethnicity (i.e., Chinese Han). ^†^There were five missing values in Hukou; these missing values were replaced by the mode of Hukou (i.e., Outlander).

**TABLE 3:** Comparison of self-report IRI-PD between subgroups of five demographic categories.

	Available and missing			Descriptive statistics for subgroups			Results of *t*-test/ANOVA				
			
Demographic	*N*	Missing	Replacing	Subgroups (*n*)	*M*	*SD*	df	*t*/*F*	*p*	*d*/η^2^	95%CI
Sex	592	0.0%	*NA*	(1) Female (386)	9.46	4.20	590	2.92	0.004	0.25	[0.34, 1.75]
				(2) Male (206)	8.41	4.08					
Ethnicity*	591	0.2%	By mode	(1) Chinese Han (489)	9.12	4.24	590	0.35	0.729	0.04	[−0.73, 1.05]
				(2) Minorities (103)	8.96	3.91					
Study major	592	0.0%	*NA*	(1) Medical (389)	8.67	4.18	590	–3.45	0.001	–0.30	[−1.95, −0.54]
				(2) Non-medical (203)	9.91	4.08					
Hukou^†^	587	0.8%	By mode	(1) Local (236)	9.29	4.27	590	0.95	0.341	0.08	[−0.36, 1.02]
				(2) Outlander (356)	8.96	4.13					
Study grade	592	0.0%	*NA*	(1) Freshman (192)	8.30	4.19	589	6.26	0.002	0.02	/
				(2) Sophomore (218)	9.75	4.19					
				(3) Senior (182)	9.14	4.06					

IRI, Interpersonal Reactivity Index; IRI-PD, the total score for IRI personal distress items. Hukou, a unique social term of China, records the administrative region of a Chinese’s original permanent address. For the Study grade, Freshman represents the newly entered and Grade 1 college students, Sophomore represents Grades 2 and 3 college students, and Senior represents Grades 4 and 5 college students. *There was one missing value in the Ethnicity; this missing value was replaced by the mode of ethnicity (i.e., Chinese Han). ^†^There were five missing values in Hukou; these missing values were replaced by the mode of Hukou (i.e., Outlander).

For IRI-PT (see Table 1), results of independent samples *t*-tests revealed a significant group difference between two study major groups (i.e., medical students < non-medical students, *t* = −4.10, *p* < 0.001, Cohen’s *d* = −0.36, 95% CI = [−1.90, −0.67]). For IRI-EC (see Table 2), significant differences were observed between two sex groups (i.e., females > males, *t* = 2.18, *p* = 0.030, Cohen’s *d* = 0.19, 95% CI = [0.06, 1.20]), two study major groups (i.e., medical students > non-medical students, *t* = 2.11, *p* = 0.035, Cohen’s *d* = 0.18, 95% CI = [0.04, 1.18]), and two Hukou groups (i.e., locals < outlanders, *t* = −2.68, *p* = 0.008, Cohen’s *d* = −0.22, 95% CI = [−1.30, −0.20]). For IRI-PD (see Table 3), significant differences were found between two sex groups (i.e., females > males, *t* = 2.92, *p* = 0.004, Cohen’s *d* = 0.25, 95% CI = [0.34, 1.75]) and two study major groups (i.e., medical students < non-medical students, *t* = −3.45, *p* = 0.001, Cohen’s *d* = −0.30, 95% CI = [−1.95, −0.54]).

One-way ANOVA analyses suggested no significant group differences between the three study grades on IRI-PT or IRI-EC (see Tables 1, 2, respectively), but there was a significant group difference in IRI-PD [*F*(1,589) = 6.26, *p* = 0.002, η^2^ = 0.02; see Table 3]. *Post-hoc* analyses further revealed that the *Freshman* group had a lower score on IRI-PD than the *Sophomore* (*p* < 0.001, Cohen’s *d* = −0.35, 95% CI = [−2.26, −0.64]) and the *Senior* groups (*p* = 0.049, Cohen’s *d* = −0.20, 95% CI = [−1.69, 0.00]). Nevertheless, between the *Sophomore* and *Senior* groups, no significant group difference in IRI-PD was found (*p* = 0.147, Cohen’s *d* = 0.15, 95% CI = [−0.21, 1.42]).

### Correlations Between Self-Reported Scores

Pearson correlation coefficients between scores on IRI subscales (i.e., IRI-PT, IRI-EC, and IRI-PD) and other self-reported questionnaires (i.e., STAI-SAI, STAI-TAI, BDI, ASLEC-SI, and ASLEC-AS) were summarized in Table 4. Results showed significant positive correlations with a medium coefficient between IRI-PD and four other scores, namely, STAI-TAI, BDI, ASLEC-SI, and ASLEC-AS (Pearson *r* range = 0.28–0.35, all *p* < 0.001). Although the correlation between IRI-PD and STAI-SAI was also positive and significant, it was with a small coefficient (Pearson *r* = 0.17, *p* < 0.001). In contrast, the Pearson correlation coefficients between IRI-PT and the other variables, as well as those for IRI-EC, were all less than 0.12 (*p*s ≥ 0.008). Cronbach’s αs for the above scales are presented in Table 4.

**TABLE 4:** Pearson correlations between self-report empathy and empathy-related scores.

	IRI-PT	IRI-EC	IRI-PD	*df*	Cronbach’s α
STAI-SAI	−0.08	−0.08	0.17*	590	0.91
STAI-TAI	−0.05	−0.05	0.35*	590	0.87
BDI-log	0.03	−0.05	0.31*	590	0.86
ASLEC-SI	0.08	0.12	0.28*	462	0.79
ASLEC-AS	0.10	0.10	0.29*	462	0.63
Cronbach’s α	0.77	0.53	0.79	/	/

IRI, Interpersonal Reactivity Index; IRI-PT, the total score for IRI perspective-taking items; IRI-EC, the total score for IRI empathic concern items; IRI-PD, the total score for IRI personal distress items; STAI, State-Trait Anxiety Inventory; STAI-SAI, the total score for STAI state anxiety items; STAI-TAI, the total score for STAI trait anxiety items; BDI, Beck Depression Inventory; BDI-log was the logarithm of the total score for BDI items; ASLEC, Adolescent Self-rating Life Event Checklist; ASLEC-SI, the total score for ASLEC social interaction items; ASLEC-AS, the total score for ASLEC academic stress items. Cronbach’s α for each scale was presented. *p < 0.001.

### The Classification Tree

#### IRI-PT (Figure 1)

For the overall students, it was found that the most powerful discriminator for IRI-PT was the study major, namely, medical students reported less IRI-PT than non-medical students (Classification Improvement = 0.37). Furthermore, the non-medical students could be further divided according to their study grade (*Freshman* vs. *Sophomore* and *Senior*; Classification Improvement = 0.48). That is, the non-medical *freshmen* reported less IRI-PT than the non-medical *sophomores* and *seniors*. The overall Risk of the Classification Tree for IRI-PT was 12.56 (*SE* = 0.70).

**FIGURE 1:**
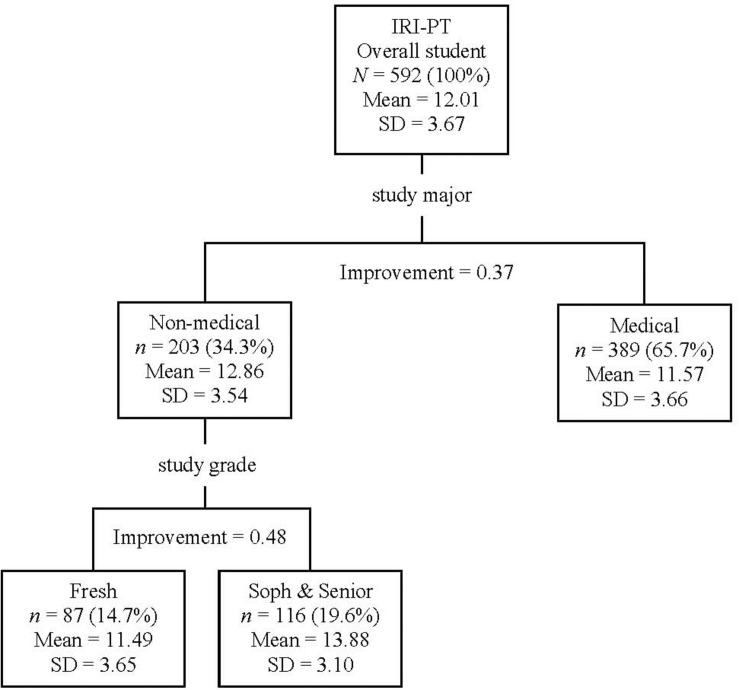
Classification tree for IRI-PT according to participants’ demographic information (i.e., study major and study grade). In the study major, students were divided into medical and non-medical students. In the study grade, *Fresh* (*Freshman*) represents the newly entered and Grade 1 college students, *Soph* (*Sophomore*) represents Grades 2 and 3 college students, and *Senior* represents Grades 4 and 5 college students. IRI, Interpersonal Reactivity Index; IRI-PT, the total score for IRI perspective-taking items.

#### IRI-EC (Figure 2)

The most powerful discriminator on the Classification Tree of IRI-EC was Hukou (Classification Improvement = 0.14). It was found that students with a local Hukou had lower IRI-EC than students with an outlander Hukou. For the branch of locals, students could be further classified into two groups according to sex (i.e., females > males, Classification Improvement = 0.13). In contrast, outlanders could be further classified according to age (i.e., age ≤ 21.5 or > 21.5); namely, younger outlander students (≤ 21.5) reported less IRI-EC than older outlander students (> 21.5; Classification Improvement = 0.11). In addition, the younger outlander students could be further divided according to their ethnicity (i.e., the Chinese Hans vs. the Chinese minorities); that is, these younger outlander students of Chinese Han had less IRI-EC than those of Chinese minorities (Classification Improvement = 0.04). The overall Risk of the Classification Tree for IRI-EC was 10.86 (*SE* = 0.61).

**FIGURE 2:**
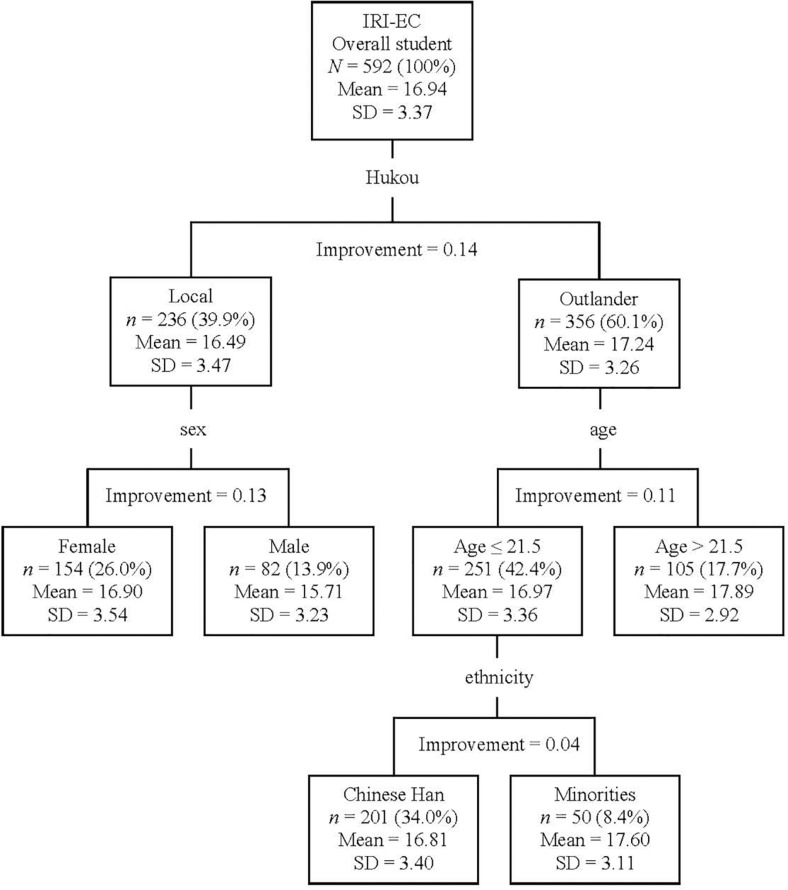
Classification tree for IRI-EC according to participants’ demographic information (i.e., Hukou, sex, age, and ethnicity). Hukou, a unique social term of China, records the administrative region of a Chinese’s original permanent address. Based on students’ Hukou and the address of their university, students were divided into locals and outlanders. According to ethnicity, students were divided into the majority (i.e., Chinese Han) and minorities of Mainland Chinese. In the current sample, there were 17 groups of ethnic minorities (e.g., Manchu and Mongolian). IRI, Interpersonal Reactivity Index; IRI-EC, the total score for IRI empathic concern items.

#### IRI-PD (Figure 3)

The most powerful discriminator on the Classification Tree of IRI-PD was the study major; that is, non-medical students reported more IRI-PD than medical students (Classification Improvement = 0.35). Moreover, the non-medical students were further dichotomized according to their study grade (*Freshman* vs. *Sophomore* and *Senior*); that is, the IRI-PD was lower for the non-medical *freshmen* than non-medical *sophomores* and *seniors* (Classification Improvement = 0.56). In contrast, medical students were further divided according to sex (females > males; Classification Improvement = 0.13). Furthermore, female medical students could be further divided according to their study grade (*Sophomore* vs. *Freshman* and *Senior*). That is, IRI-PD was found to be higher for the female *sophomores* of medical students than female *freshmen* and *seniors* of medical students (Classification Improvement = 0.14). The overall Risk of the Classification Tree for IRI-PD was 16.31 (*SE* = 0.88).

**FIGURE 3:**
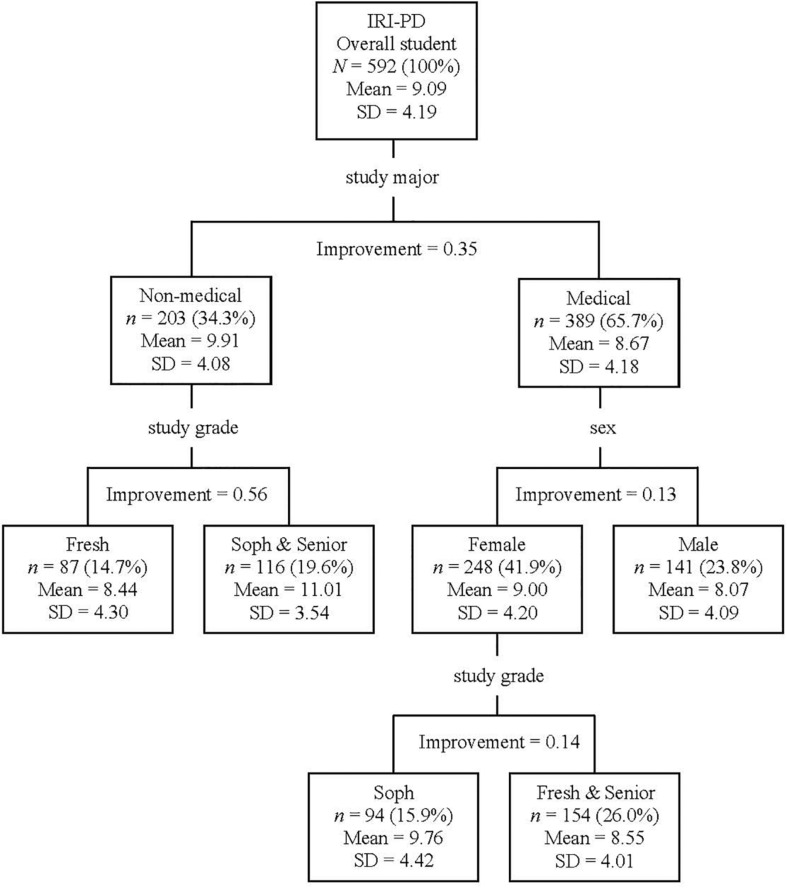
Classification tree for IRI-PD according to participants’ demographic information (i.e., study major, study grade, and sex). In the study major, *Fresh* (*Freshman*) represents the newly entered and Grade 1 college students, *Soph* (*Sophomore*) represents Grades 2 and 3 college students, and *Senior* represents Grades 4 and 5 college students. IRI, Interpersonal Reactivity Index; IRI-PD, the total score for IRI personal distress items.

## Discussion

In the current study, self-report empathy and related characteristics were examined in a cohort of Mainland Chinese youth. It was found that the impact factors for emotional empathy were inherited traits (e.g., sex); for cognitive empathy were acquired traits (e.g., study major); and for personal distress were the combination of inherited and acquired traits (e.g., sex and study major). Meanwhile, emotional empathy was higher for youth with a vulnerable trait (i.e., outlander, female, and ethnic minority) than those with a dominant one (i.e., local, male, and ethnic majority). Regarding the study major, no evidence supported the concern that medical training reduces empathy; instead, it was found that the non-medical course promoted cognitive empathy. Moreover, it was observed that students’ wellness was closely related to their self-report personal distress, rather than empathy *per se*.

The current results suggested that the impact factors differed for emotional and cognitive empathy. The CART analyses (see Figures 1, 2) suggested that inherited traits (i.e., Hukou, sex, age, and ethnicity) impacted emotional empathy, while acquired traits (i.e., study major and study grade) impacted cognitive empathy. Emotional empathy is an automatic response to other’s emotions and is the primary form of empathy; in contrast, cognitive empathy is an advanced form of empathy, requiring cognitive processing (Shamay-Tsoory et al., 2009). Therefore, the current results might suggest that cognitive empathy could be more malleable than emotional empathy, which ought to be considered by future educators and trainers aiming at promoting empathy in youth. According to Shamay-Tsoory et al. (2009), the key development period for emotional empathy is infancy, while the one for cognitive empathy lasts up to childhood and adolescence. The current findings were in line with the aforementioned understanding of empathy and also provided new evidence supporting the dissociation of the two concepts (see Shamay-Tsoory et al., 2009).

In the aspect of emotional empathy, youth in vulnerable positions, namely, female (i.e., sex), minority (i.e., ethnicity), and outlander (i.e., Hukou), reported a higher score than their counterparts in dominant positions (i.e., males, the ethnic majority, and locals, respectively). This was a new observation with Mainland Chinese youth. Interestingly, Teague (2014) observed parallel findings with American participants. Firstly, they observed that female Americans had higher self-report emotional empathy than male Americans from three ethnicities (viz, Caucasian, African, and Asian) (Teague, 2014). Secondly, they found that African and Asian Americans were better at recognizing the emotions of Caucasians than their own ethnic groups, but such out-group favor was not shown by the Caucasian Americans (Teague, 2014). One reasonable consideration is that individuals in a more vulnerable position received more social pressure to be alert of others’ changing moods. Consequently, individuals with a more vulnerable position might have higher emotional empathy, as reflected in the current study. Further studies might consider directly examining the subjective evaluation of vulnerability, social expectation, and empathy to empirically examine this topic.

Sex was not the most important impact factor of empathy with the current Mainland Chinese youth. With German and Ethiopian students, Dehning et al. (2013) identified that sex was the most important impact factor of self-report emotional empathy (i.e., females > males). Nevertheless, previous researchers have pointed out that the sex difference in self-report empathy in Mainland Chinese participants (Zhao et al., 2018) was not as significant as reported in Western populations (e.g., Groen et al., 2015). It was considered that the decreased sex differences in empathy in Mainland Chinese could be due to the Confucius’ Golden Mean philosophy, which requires people to behave between two extremes (e.g., neither be extremely masculine nor extremely feminine) (Zhao et al., 2019).

To date, whether medical training has a positive (e.g., Penprase et al., 2013) or negative impact (e.g., Nunes et al., 2011) on empathy is still under debate. In the current study, the study major was found to be the most important impact factor of cognitive empathy. It was found that the overall medical students (from newly entered to senior students) had less cognitive empathy than their overall non-medical counterpart. However, this difference should not be blamed on the medical training *per se* as no evidence was found in the current study suggesting a direct negative influence from medical training on cognitive empathy in the current Mainland Chinese youth. In contrast, after the 1st year of college training, there was an increase in cognitive empathy in non-medical students, but this increase was absent in medical students. On the one hand, the aforementioned difference might reflect a trait of Mainland Chinese students who choose to take the medical course. On the other hand, the difference might be in line with a theory mentioned by Dehning et al. (2013); that is, whether the medical course has a so-called negative impact on students’ empathy may depend on whether the curriculum includes humanities and art courses (Kataoka et al., 2009).

Interestingly, the current results suggested that although medical students had less cognitive empathy than non-medical students, this disadvantage was counterbalanced by the fact that the latter group suffered more personal distress (i.e., a self-oriented automatic aversive response to other’s suffering) than the former group. It should be noticed that high personal distress could cause individuals to avoid cognitive empathy for other’s suffering (e.g., sadness or injuries) to protect themselves from emotional exhaustion (López-Pérez et al., 2014). Nonetheless, this type of behavior contradicts the Hippocratic Oath “I will apply dietetic measures for the benefit of the sick according to my ability and judgment; I will keep them from harm and injustice.” (Edelstein, 2000, p. 3). In the current study, it was found that the overall medical students self-reported less personal distress than non-medical students for empathy. Moreover, after the 1st-year college training, there was an increase in personal distress in non-medical students, while only a fluctuation in personal distress was observed in female medical students during Grades 2–3. Thereafter, according to Dehning et al. (2013) and Kataoka et al. (2009), including humanities or art courses may be helpful for medical students to increase cognitive empathy, but it is unknown whether this action would also incur an increase of personal distress.

It was also noticeable that both the impact factors of emotional and cognitive empathy (i.e., sex, study major, and study grade) had an impact on personal distress (see Figure 3). Personal distress has an intricate relationship with empathy. Some researchers deemed personal distress as a type of emotional empathy (e.g., Davis, 1980), while others stated it was an independent concept (e.g., Baron-Cohen and Wheelwright, 2004). Researchers frequently found that self-report personal distress and emotional empathy were positively correlated, but also observed a negative correlation between personal distress and cognitive empathy (e.g., Zhao et al., 2019). Recently, a group of researchers considered that personal distress may have negative feedback on the later stage of empathy (i.e., cognitive empathy), but not on the automatic stage of empathy (i.e., emotional empathy) (Zhao et al., 2019). Furthermore, in the current study, it was found that students’ wellness (i.e., depression, anxiety, and problems in academic and social activities) was closely related to personal distress, but not empathy *per se*. Moreover, a cross-cultural study suggested that both male and female Mainland Chinese youth suffered more personal distress than their Australian counterparts (Zhao et al., 2019). Therefore, managing personal distress could be an important goal for Mainland Chinese youth.

The current study has several limitations. First, this study was based on a convenient sample of university students. Therefore, the current results might not represent the trait of the overall Mainland Chinese youth. Second, participants of this study were recruited only from four universities, while the conclusion regarding the relationship between empathy and both demographic information and personal characteristics requires further investigation based on a random selection of universities from cities around Mainland China. Third, several demographic information (e.g., family income and urban/rural dwellers) might also have an impact on youth’s empathy but was not covered in this study. Instead, we focused on information that is presented on the national ID and student cards (e.g., ethnicity and study major), and tried to avoid collecting information that might put some youths out of their comfort zone or make them feel self-abased. Fourth, the current study focused on empathy for emotions (measured by IRI scores), while empathy for pain (e.g., social and physical pain; see Zhang et al., 2019) is also an important social and medical skill (see Gu et al., 2019). A future investigation of empathy for pain in Mainland Chinese youth, for a cross-study comparison with the current findings, would be worthwhile. Fifth, it should be noticed that the current study was a cross-sectional investigation, but a longitudinal observation is better to portray the trajectory of empathy development in youth. Finally, the current results were based on participants’ self-evaluations, which could be criticized as subjective compared to behavioral or brain imaging investigations. The latter techniques should be involved in further investigation of the current topic.

## Conclusion

The current authors investigated empathy and its impact factors in a cohort of Mainland Chinese youth. Results suggested that youth’s inherited and acquired traits impacted emotional and cognitive empathy, respectively. In the aspect of emotional empathy, it was found that youth in a more vulnerable position showed more emotional empathy than their dominant counterparts. In the aspect of cognitive empathy, the trajectory of its development was with an increase in the non-medical students after 1-year college training, but this increase was absent with the medical students. This result implied that cognitive empathy might be more malleable than emotional empathy; therefore, curriculum designers who aim to enhance youth empathy might consider starting from cognitive empathy-related training (e.g., how to appreciate other’s feelings through role-taking). Finally, it was found that personal distress, rather than empathy, was significantly correlated with anxiety and depression, as well as academic and social problems in youth. The current findings provided a fresh understanding of the impact factors of empathy in youth, and particularly, it called attention to the importance of personal distress-management to promote the well-being of Mainland Chinese youth.

## Data Availability Statement

The datasets generated for this study are available on request to the last corresponding author.

## Ethics Statement

The study involving human participants was reviewed and approved by the Institute of Psychology, Chinese Academy of Sciences. Written informed consent from the participants’ legal guardian/next of kin was not required to participate in this study in accordance with the national legislation and the institutional requirements.

## Author Contributions

QZ conducted the analyses and wrote the manuscript. QR conducted the pre-analyses and participated in the manuscript writing. YS collected the data and participated in the manuscript writing. LW participated in the study design and manuscript writing. LH designed the research and wrote the manuscript.

## Conflict of Interest

The authors declare that the research was conducted in the absence of any commercial or financial relationships that could be construed as a potential conflict of interest.

## Footnotes

1 www.statulator.com
